# The Prevalence of Anxiety among Men Who Have Sex with Men: A Protocol for A Systematic Review and Meta-Analysis

**DOI:** 10.3390/ijerph20021315

**Published:** 2023-01-11

**Authors:** Jinhong Yang, Zemiao Zhang, Ling Jie Cheng, Jia Chen, Vivien Xi Wu

**Affiliations:** 1 Xiangya School of Nursing, Central South University, Changsha 410013, China; 2 Key Laboratory of Nursing of Hunan Province, Changsha 410013, China; 3 School of Medicine and Health Management, Tongji Medical College, Huazhong University of Science and Technology, Wuhan 430030, China; 4 Saw Swee Hock School of Public Health, National University of Singapore, Singapore 117549, Singapore; 5 Alice Lee Centre for Nursing Studies, Yong Loo Lin School of Medicine, National University of Singapore, Singapore 117597, Singapore

**Keywords:** anxiety, men who have sex with men, prevalence, protocol

## Abstract

Anxiety is reported to be common and serious among men who have sex with men (MSM). A growing number of researchers focus on MSM’s anxiety and reported their severity, while the estimate results vary substantially between studies. The objective of this study is to evaluate the pooled prevalence of anxiety or anxiety disorders among MSM worldwide. This systematic review and meta-analysis protocol will follow the Preferred Reporting Items for Systematic Reviews and Meta-Analysis (PRISMA-P) guidelines. Studies will be searched from English and Chinese electronic databases. Observational studies, longitudinal studies, and controlled trials reporting the prevalence of anxiety or anxiety disorders among MSM will be included. Two reviewers will independently screen all the studies and extract data. Quality appraisal will be conducted using the Joanna Briggs Institute’s (JBI’s) critical appraisal checklist for reporting prevalence data. Meta-analysis will be implemented with a random-effect model which will evaluate pooled prevalence of anxiety with 95% confidence intervals (CI). Subgroup analysis will be conducted among different regions, sampling methods, data collection methods, MSM characteristics, measures used to assess anxiety and its cut-off. This review will contribute to a great understanding of anxiety among MSM worldwide. The findings will help relative policymakers and researchers develop effective measures and interventions for reducing the burden of anxiety morbidity among this population.

## 1. Introduction

Men who have sex with men (MSM) are males who engage in sexual activity with members of the same biological gender, regardless of how they identify themselves. MSM accounted for 3.8% to 6.4% of men in the US, and the proportion peaked at 6.5% in European countries [1]. MSM constitute a certain proportion of the male population and is worth to concern, despite being a minority group.

Although society is becoming more open-minded ad accepting of sexual minorities [2], such as the gay liberation movement and the decriminalization of homosexuality in some countries in recent years, they continue to face far more stigma, prejudice, and discrimination, whether from individuals or society. Minority stress theory holds that social stressors related to stigma and prejudice cause mental health disorders, such as depression and anxiety [3]. Anxiety is an emotion characterized by tension, worried thoughts, and physical changes, such as anxiety disorders are excessive fear or anxiety. Types of anxiety disorders generally involve generalized anxiety disorder, panic disorder, phobias, agoraphobia, and social anxiety disorder. Since MSM are a sexual minority, the pressure from homosexuality-related stigma and discrimination may contribute to an increased risk of anxiety. A longitudinal study revealed that self-stigma, which is part of internalized stigma, is associated with symptoms of generalized anxiety disorder and is positively predicted in MSM [4]. Furthermore, the lack of social acceptance and social support may indirectly influence MSM to develop weak self-cognition and self-acceptance. The hostile and stressful social environment for sexual minorities easily traps them in self-doubt, leading MSM to have a higher prevalence of mental health problems than heterosexuals [3]. Several studies demonstrated that the risk of mental health disorders, including anxiety, in sexual minorities was 1.5 to 3 times higher than in sexual majorities [5,6]. In addition, differences in religious beliefs or policies lead to different degrees of social acceptance of MSM in different countries or regions, making their anxiety or anxiety disorders severity vary from region to region.

Due to the sexual behavior related characteristics of this population, high-risk sexual behavior, the primary cause of HIV transmission through sexual contact, frequently occurs among MSM. Unprotected anal intercourse (UAI), sex with multiple partners, and commercial sex activity are examples of high-risk sexual behavior [7]. Therefore, compared to heterosexual males, MSM have a higher risk and probability of being infected with HIV and other sexually transmitted diseases (STDs). The risk of acquiring HIV among MSM worldwide is 26 times higher than in the general population [8]. According to a systematic review, the average HIV prevalence among MSM and the general male population in Sub-Saharan Africa was 17.81% and 6.15%, respectively [9]. Hence, this increased susceptibility to HIV infection makes MSM susceptible to mental health problems. According to a survey conducted in Australia, the prevalence of anxiety in HIV-positive MSM was much higher than in national men, at 36% versus 11%, and HIV-related discrimination and being unfairly treated due to having HIV predicted their severity of anxiety [10].

Overall, the state of anxiety among MSM is significant to research. Numerous researchers were aware of it and conducted related studies to estimate the prevalence of anxiety among MSM. An assessment of reliable anxiety prevalence estimates is the first and essential step to identifying the status of this population and assisting public health professionals in developing and implementing effective strategies for MSM across different ethnicities and countries. Despite an influx of studies focusing on the prevalence of anxiety among MSM, the results have varied greatly, ranging from 12.7% to 57.58% [11,12]. To date, only one meta-analysis has reported a pooled prevalence of anxiety among MSM, at 32.3%, and it was limited to MSM in China [13]. Therefore, to address the literature gap, we aim to determine the prevalence of anxiety in MSM worldwide using meta-analysis and exploring sources of heterogeneity using subgroup and meta-regression. We hope the results of this systematic review will provide accurate and convictive data to related policy-makers and lead them to be conscious of the severity of MSM’s anxiety issues and incorporate measures to reduce anxiety or anxiety disorders into overall intervention strategies for MSM.

## 2. Method

This systematic review was registered in the PROSPERO database (Registration number: CRD42022356307), and will be implemented in accordance with the standards of Preferred Reporting Items for Systematic Reviews and Meta-analysis (PRISMA) guidelines [14] and Meta-analysis of Observational Studies in Epidemiology (MOOSE) guidelines [15]. This protocol follows the PRISMA-P guidelines [16,17].

### 2.1. Information Sources and Search Strategy

Two reviewers (YJH and ZZM) will search studies in six English electronic databases: PubMed, Web of Science, Embase, MEDLINE (Ovid interface), the Cochrane Library, and PsycINFO. Two Chinese databases (Chinses National Knowledge Infrastructure (CNKI) and Wanfang database) will be searched. No date restrictions will be applied on the search. The search strategy will combine specific search terms (e.g., MeSH terms) for each database and free terms for two concepts: “Men who have Sex with Men” AND (“anxiety” OR “anxiety disorders”). We display a full search strategy for one database in Appendix A. We will also examine reference lists of included articles to search for studies that meet the eligibility criteria.

### 2.2. Eligibility Criteria

Studies will be included based on following types of criteria: study design, participants, and outcomes.

#### 2.2.1. Types of Studies

Observational studies such as cross-sectional, cohort, case-control studies which reporting the prevalence of anxiety or anxiety disorders among MSM will be included. If any longitudinal studies and controlled trails are identified that report relevant data, the baseline data will be collected. Papers searched from both English and Chinese will be included. Qualitative studies, commentary, review, and case series etc. will be excluded. If multiple papers used the same dataset, only one paper with complete data will be selected.

#### 2.2.2. Types of Participants

Studies targeted at MSM, including gay and bisexual men, will be considered. Whereas studies whose population contained MSM but did not report their prevalence of anxiety or anxiety disorders will be excluded.

#### 2.2.3. Types of Outcomes

The main outcome of the systematic review will be the prevalence of anxiety or anxiety disorders indicating the number of MSM with anxiety or anxiety disorders symptom divided by the total number of MSM in a specific time. This is often presented as a prevalence. Moreover, studies used a standardized measurement such as diagnostic scale (e.g., Generalized Anxiety Disorder Scale, the Anxiety Self-Rating Scale) or structured clinical interview (e.g., Structured Clinical Interview for DSM-IV) to assess anxiety or anxiety disorders will be selected.

### 2.3. Study Selection

**Figure 1:**
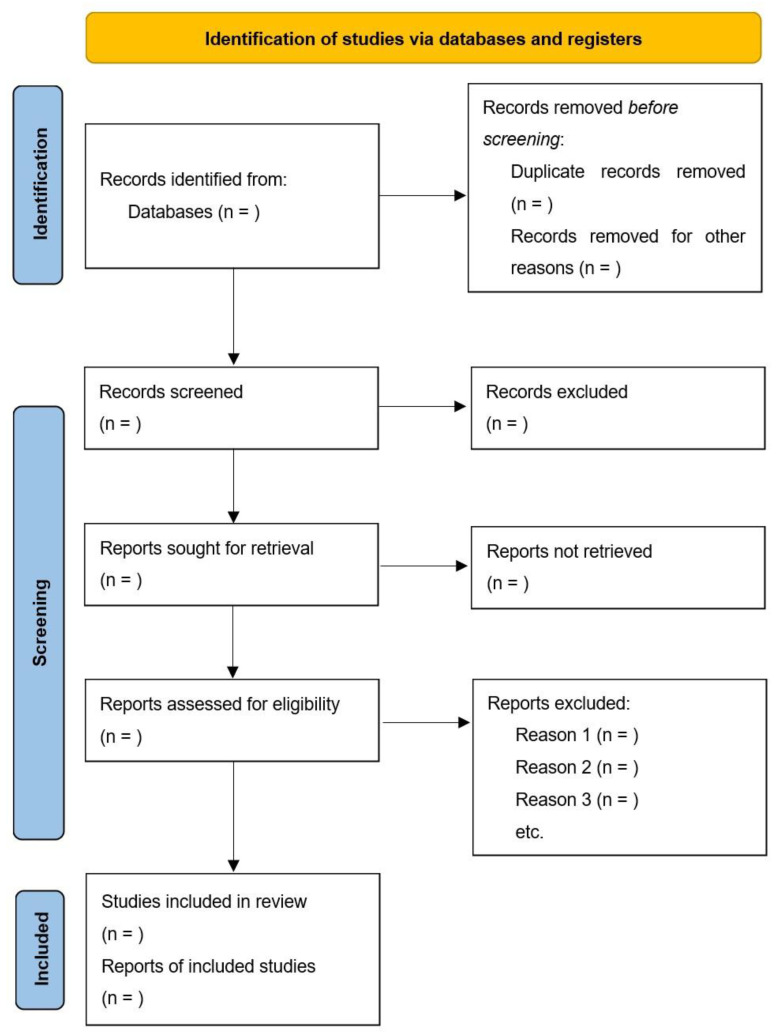
PRISMA 2020 Flow Diagram for New Systematic Reviews.

All studies searched from databases will be screened by two reviewers (YJH and ZZM) and managed in EndNote [18]. After removing duplicate automatically by EndNote, two reviewers will independently screen all titles and abstracts based on inclusion and exclusion criteria, to identify initial included studies. The full texts of these studies will be further screened in the same way. If full text cannot be obtained, we will ask the university librarian for help in obtaining the full text. Any unreached consensus or conflict will be resolved by the third reviewer (VXW). A PRISMA flow chart (Figure 1) will be used to show the details of the study selection process.

### 2.4. Data Extraction

Two reviewers will separately conduct data extraction. The third reviewer will provide suggestion and solution to any inconsistencies. The data to be extracted will be: (1) Study Data: first author’s surname, publication year, survey dates, location and country, setting, design method, sampling method, data collection method; (2) Participant Data: population type (e.g., HIV-positive MSM, HIV-negative MSM), sample size, age (e.g., Mean with SD, Median, or Range); (3) Outcome Data: measure used to assess anxiety and its cut-off value, and prevalence of anxiety or anxiety disorders among MSM. If information is missing or couldn’t be retrieved in the full text, we will contact their corresponding authors to obtain.

### 2.5. Quality Assessment

The Joanna Briggs Institute’s (JBI’s) critical appraisal checklist for studies reporting prevalence data [19] will be selected to assess the quality of the included studies by two reviewers independently. Any inconsistencies will be resolved by discussion with the third reviewer. These checklists contain “Yes”, “No”, “Unclear” and “Not Applicable” response options. “Yes” is scored “1”, while “No”, “Unclear” or “Not Applicable” is scored “0”. The total score is the sum of the scores of each item, and the higher the total score the higher the quality of the study. Studies will be included regardless of their quality, but this will be considered in the final discussion.

### 2.6. Data Analysis

All analysis will be conducted using STATA-17 software. If possible and appropriate, the meta-analysis of prevalence of anxiety among MSM will be conducted using a random-effect model which will generate pooled prevalence estimates with 95% confidence intervals (CIs) [20]. The weighted prevalence will be calculated by the Logit transformation way to improve their statistical properties. Heterogeneity will be determined using the Cochran’s Q test and I-squared test, *p* < 0.05 and I^2^ ≥ 50% indicating significant heterogeneity [21,22]. We will use forest plots to visualize the pooled prevalence estimates and the extent of heterogeneity. Subgroups analysis and meta-regression will be used to estimate the influence of various covariates on the pooled prevalence of anxiety and conducted to explore the source of heterogeneity. The pre-defined covariates that will be used in subgroup analysis includes region, sampling method, data collection method, MSM characteristics, measure used to assess anxiety and its cut-off. The covariates that will be modelled in the meta-regression are survey year, year of publication, and age. We will use the bubble plot to graphically illustrate which covariate will be associated with the prevalence of anxiety and to present the effect of this covariate on the level of anxiety [23]. If more than 10 studies are pooled in meta-analysis, the publication bias will be detected by visual inspection of the funnel plot and Egger’s regression test, *p* < 0.05 shows statistically significant [24,25]. 

## 3. Discussion

As one of the most common mental health disorders, anxiety or anxiety disorders are prevalent and serious in the MSM population. Understanding the overall situation of the prevalence estimate of anxiety is the first and essential step for public health researchers in developing strategies and conducting interventions. This systematic review and meta-analysis will discuss and provide pooled prevalence estimates of anxiety or anxiety disorders among MSM worldwide, reflect on the situation and severity intuitively, and then encourage psychologists and researchers pay greater attention to MSM’s anxiety. Hence, we hope this systematic review could offer high-quality evidence for policy-level decision-making, which help decision-makers develop targeted systematic interventions and policies in their region, and contribute to reducing the incidence of anxiety among MSM. Moreover, the prior pooled evidence on the anxiety status of MSM could assist policymakers by allocating appropriate resources to relevant epidemiological or experimental studies, further prompting more professionals and institutions to research and implement mental health interventions for MSM.

This review will focus only on the anxiety status among MSM and explore the sources of heterogeneity by subgroup analysis and meta-regression. This systematic review will be predicted to have heterogeneity, as the included studies involve global populations, which may result in different outcomes for each region or race. In addition, different characteristics of MSM and different measurements may affect anxiety prevalence. As we will search and include only published literature, publication bias will potentially exist. We will assess and visualize this bias. Another limitation of this review is that articles written in languages other than English and Chinese may be missed, due to the practical reason which is the language limitations of the reviewers. Despite potential limitations, the results and information from this review are likely to affect mental health intervention and treatment among sexual minorities positively.

## 4. Conclusions

This study facilitates the protocol methods for a systematic review that explores the pooled prevalence of anxiety or anxiety disorders among MSM worldwide. The results from this systematic review and meta-analysis will be useful for researchers and decision-makers to implement interventions and improve anxiety health questions in MSM. The systematic review and meta-analysis will be strictly implemented according to the guidance provided in the PRISMA 2020 statement and MOOSE guidelines. The actual conduct and results will be displayed in the final report, which plan to be published in a peer-reviewed journal.

## Data Availability

No data were created.

## Appendix A. Example of the Search Strategy Used in PubMed

**Search Number**	**Search Strategy**
#1	“Homosexuality, Male”[Mesh] OR “Sexual and Gender Minorities”[Mesh]
#2	“Men Who Have Sex with Men”[Title/Abstract] OR MSM[Title/Abstract] OR Gay*[Title/Abstract] OR Homosexual*[Title/Abstract] OR “Male Homosexuality”[Title/Abstract] OR Bisexual*[Title/Abstract] OR Queer*[Title/Abstract] OR “Gender Minorit*”[Title/Abstract] OR “Sexual Minorit*”[Title/Abstract] OR “sex between men”[Title/Abstract]
#3	“Anxiety Disorders”[Mesh] OR “Anxiety”[Mesh]
#4	Anxiety[Title/Abstract] AND (Symptom*[Title/Abstract] OR Disorder*[Title/Abstract] OR Neuroses[Title/Abstract])
#5	“Social Anxiet*”[Title/Abstract] OR “Neurotic Anxiety State*”[Title/Abstract] OR Angst[Title/Abstract] OR Hypervigilance[Title/Abstract] OR Nervousness[Title/Abstract] OR Nervous[Title/Abstract] OR Anxiousness[Title/Abstract] OR Anxious[Title/Abstract]
#6	#1 OR #2
#7	#3 OR #4 OR #5
#8	#6 AND #7
